# Colloidal particle aggregation: mechanism of assembly studied via constructal theory modeling

**DOI:** 10.3762/bjnano.12.33

**Published:** 2021-05-06

**Authors:** Scott C Bukosky, Sukrith Dev, Monica S Allen, Jeffery W Allen

**Affiliations:** 1 Air Force Research Laboratory, Munitions Directorate, Eglin AFB, FL 32542, USA

**Keywords:** colloids, constructal law, DLVO theory, interparticle interactions, nanomaterials, self-assembly, tunable systems

## Abstract

The assembly of colloidal particles into ordered structures is of great importance to a variety of nanoscale applications where the precise control and placement of particles is essential. A fundamental understanding of this assembly mechanism is necessary to not only predict, but also to tune the desired properties of a given system. Here, we use constructal theory to develop a theoretical model to explain this mechanism with respect to van der Waals and double layer interactions. Preliminary results show that the particle aggregation behavior depends on the initial lattice configuration and solvent properties. Ultimately, our model provides the first constructal framework for predicting the self-assembly of particles and could be expanded upon to fit a range of colloidal systems.

## Introduction

Constructal theory has been used to describe a number of naturally evolving processes/phenomena that include, but are not limited to, turbulent flow, heat and mass transfer, dendritic formation, and biological growth [1–10]. According to this law: “For a flow system to persist in time (to live), it must evolve in such a way that it provides easier access to the imposed (global) currents that flow through it” [1]. The main distinction between the classical laws of thermodynamics and constructal law is the former only describes the final equilibrium state of the system while the latter describes how and through what pathway(s) the system naturally progresses to achieve that final equilibrium state.

In concurrence with such constructal analyses, Bejan and Wagstaff showed that the natural coalescence of masses in space, with respect to attractive gravitational forces, will lead to a hierarchical distribution [11]. The internal tension of the system, which drives this natural coalescence, is decreased faster and more efficiently when the masses coalesce non-uniformly toward a state of equilibrium [10]. Similarly, Reis et al. used these principles to analyze how the electrostatic interactions between dust particles are minimized during particle agglomeration in air-cleaning devices [12]. It was concluded that the maximum collection rate was achieved when the aerosol particles first formed into clusters and then dendrites in order to balance their electrostatic forces. The aggregation of colloidal particles (small particles or droplets typically between 10 nm and 10 µm in diameter that can be suspended in a fluid via Brownian motion [13]) is, therefore, expected to adhere to a similar natural progression such that the total energy of the system is minimized. When analyzing colloidal systems, however, both attractive and repulsive forces must be taken into consideration.

The pairwise interactions between these particles are often described by traditional colloidal Derjaguin–Landau–Verwey–Overbeek (DLVO) theory [14]. Here, electrolyte ions in solution rearrange to form electric double layers near the charged surface of a particle. Although overlapping double layers result in repulsion between two particles, this force is constantly opposed by the attractive van der Waals force. The balance between these interparticle forces gives the total DLVO force and highly depends on system parameters, such as the electrolyte concentration and fluid dielectric constant, as well as the particle size, spacing, and surface charge. As a result, these system parameters can be tuned in order to control the balance between the DLVO forces.

Tunability, or the ability to direct the movement and assembly of particles into higher-order structures, is important for a wide variety of applications, including plasmonic/photonic devices, metamaterials, biomaterials and coatings, energetic materials, and high-strength ceramics [15–25]. Most applications, however, require precise manipulation of the particle placement, spacing, and arrangement in order to achieve desired macroscale properties. Thus, a theoretical understanding of the particle assembly mechanism is necessary to predict the nano/microscale structures responsible for the desired or observable properties.

To our knowledge, constructal theory has never been applied/implemented to describe the assembly of colloidal particles. In this article, we use said theory to predict the effect of varying system parameters on the magnitude of the total DLVO force for two scenarios, that is, uniform and non-uniform aggregation. The constructal approach presented here provides a fundamental framework for understanding and tuning the assembly behavior of colloidal particles, which could have implications across a broad range of fabrication techniques.

## Model Details

**Figure 1:**
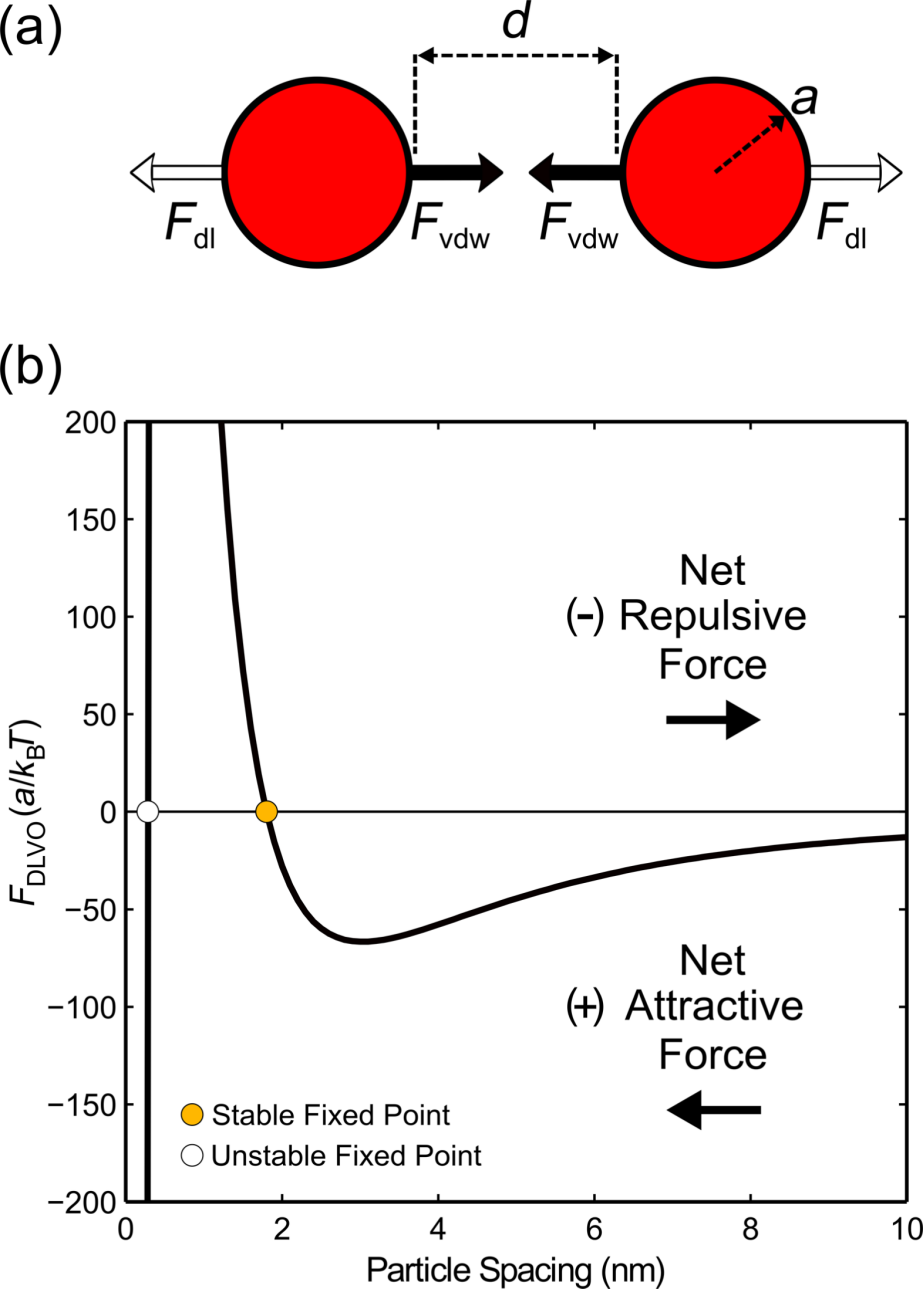
(a) Schematic of the pairwise DLVO interactions between particles (not to scale). (b) Representative total DLVO force distribution resulting from a balance between the van der Waals and double layer forces. For negatively charged particles, a net attractive force is felt when *F*_DLVO_ < 0. Markers denote the fixed point locations where the van der Waals and double layer forces are balanced (net force is zero).

First, we consider the pairwise DLVO interactions between two isolated spheres suspended in an electrolyte solution. The electrolyte ions arrange near the surface of the particles to form an electric double layer, thus screening the surface charge. The characteristic length or “thickness” of this double layer (which is a function of the ion concentration, *I*) is known as the Debye length, λ_D_, while the surface charge is parameterized by the zeta potential, ζ_p_. This repulsive double layer force is given by [14]:

[1] $$\begin{array}{l} F_{\text{dl}}\left(d\right)=Z\frac{a}{\lambda_{\text{D}}}e^{-d/\lambda_{\text{D}}};\\ Z=32\pi \varepsilon_{0}\varepsilon_{\text{c}}{\left(\frac{k_{\text{B}}T}{e}\right)}^{2}\tanh^{2}\left(\frac{e\zeta_{\text{p}}}{4k_{\text{B}}T}\right), \end{array}$$

where *d* is the particle separation distance, *a* is the particle radius, *T* is the temperature of the system, *e* is the elementary charge, *k*_B_ is Boltzmann’s constant, and ε_0_ and ε_c_ are vacuum permittivity and fluid dielectric constant, respectively. Conversely, the attractive van der Waals force is given by [14]:

[2] $$F_{\text{vdW}}\left(d\right)=\frac{-A}{12}\frac{a}{d^{2}},$$

where the characteristic energy scale is set by the Hamaker constant, *A*. It is noted that Equation 1 and Equation 2 assume spherical particles of equal radius and a sufficiently small separation distance (*a* ≫ *d*). A simplified schematic of this pairwise particle interaction is shown in Figure 1a. The resulting total DLVO force is then written as the sum of the double layer repulsion and the van der Waals attraction:

[3] $$F_{\text{DLVO}}\left(d\right)=F_{\text{dl}}+F_{\text{vdW}}.$$

When *F*_DLVO_ < 0 or *F*_DLVO_ > 0, negatively charged particles will experience a net attractive or repulsive force, respectively (Figure 1b). Therefore, when *F*_DLVO_ = 0, the particles experience zero net force. These fixed point locations are observed in Figure 1b where *F*_DLVO_ intersects the equilibrium line. The location of the stable fixed point, which varies based on the particle and solvent properties, determines the preferred interparticle separation distance. Ultimately, this DLVO framework is used to describe the structural evolution of colloidal particle assemblies.

### Aggregation in one dimension

**Figure 2:**
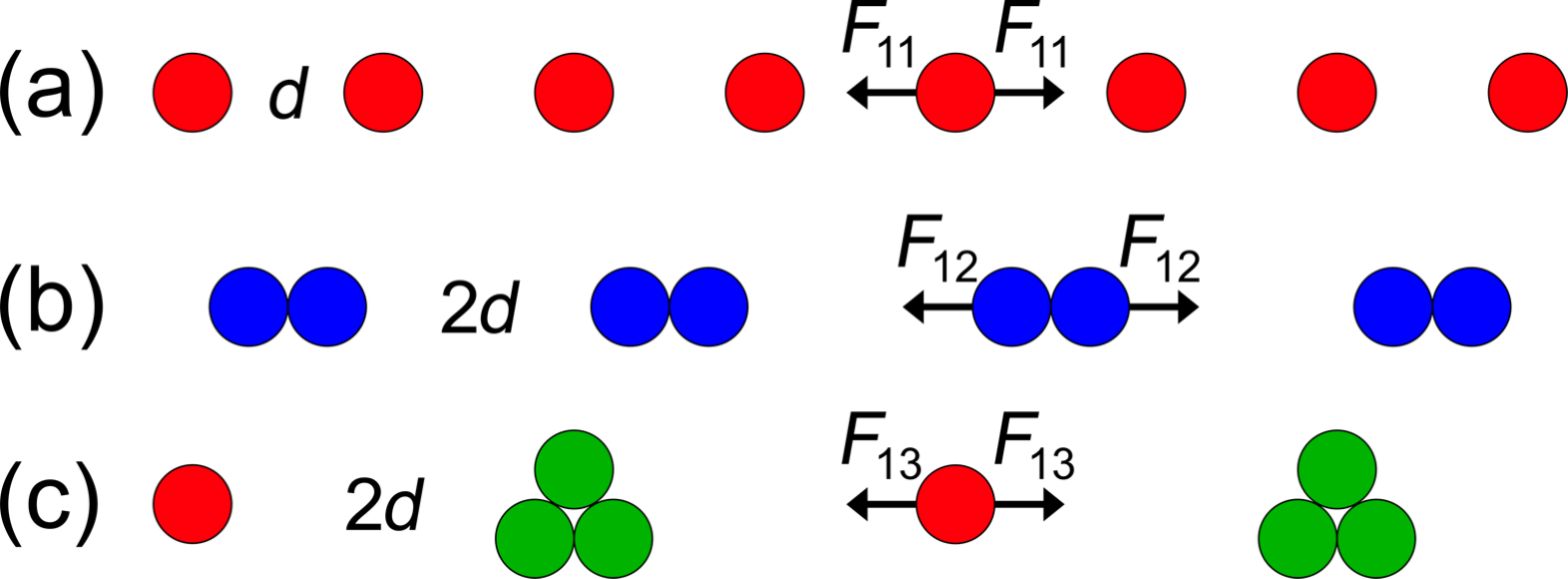
(a) 1D array of particles that undergo (b) uniform assembly or (c) non-uniform assembly.

Similar to the analysis by Bejan and Wagstaff [11], we begin by examining the one-dimensional (1D) assembly of particles. Initially, the particles are set at an evenly spaced distance (*d*) along an infinite line (Figure 2a). In this initial state each particle feels a net DLVO force, *F*_11_, in either direction due to the forces from all other neighboring particles along the line. Therefore, based on Equation 2, the total sum of the van der Waals contributions is:

[4]
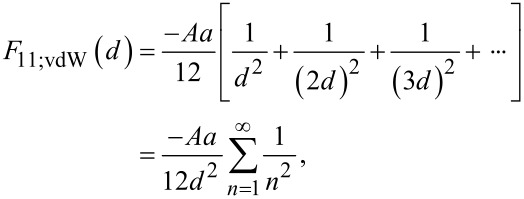


[5] $$F_{\text{11;vdW}}\left(d\right)=\frac{-Aa}{12d^{2}}\left(\frac{\pi^{2}}{6}\right).$$

Likewise, from Equation 1, the total double layer contribution is:

[6]
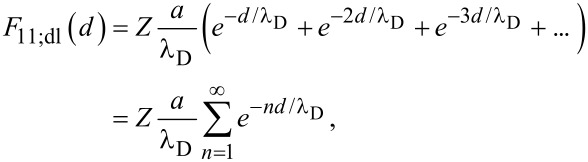


[7] $$F_{\text{11;dl}}\left(d\right)=Z\frac{a}{\lambda_{\text{D}}}\left(\frac{1}{e^{d/\lambda_{\text{D}}}-1}\right).$$

As the system begins to evolve from this initial state, the particles can assemble either via uniform or non-uniform aggregation. With respect to uniform aggregation of identical particles, two neighboring particles uniformly come together to form doublets with a new spacing of 2*d* (Figure 2b). Each particle separately feels the forces from the individual particles of the neighboring doublet, which results in an increase by a factor of four in the total force, where:

[8]
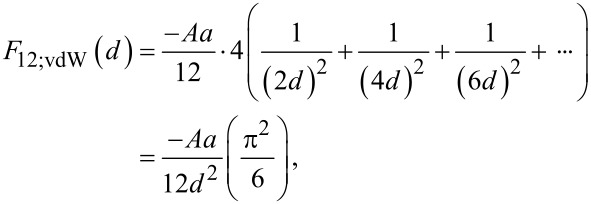


and

[9]
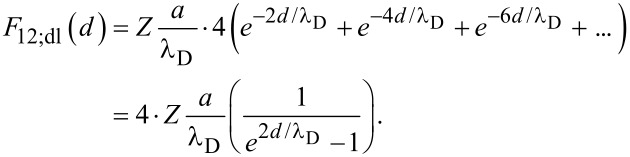


It is noted that the van der Waals force for uniform aggregation is equal to that of the initial particle configuration (i.e., *F*_11;vdW_ = *F*_12;vdW_). Therefore, the magnitude of *F*_12_ compared to *F*_11_ depends only on the double layer term.

Alternatively, with respect to a non-uniform aggregation of identical particles, two particles combine with a single neighbor (Figure 2c), which results in a cluster of three particles with one lone particle remaining. Again, the spacing between each singlet/triplet pair is 2*d*. The total force, *F*_13_, on a single particle is, therefore, the sum of the contributions from alternating clusters and singlets, where:

[10]
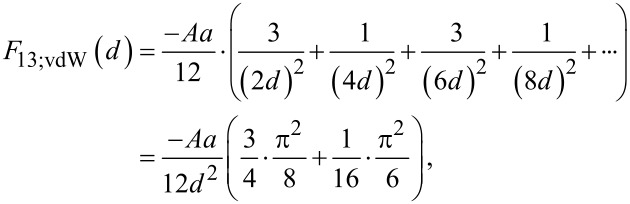


and

[11]
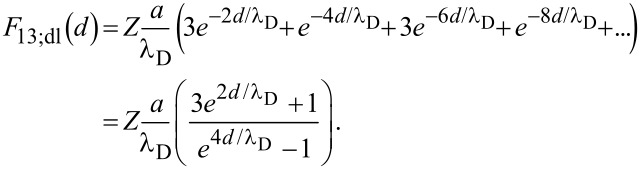


Compared to the initial particle configuration, it can be deduced from Equation 10 and Equation 11 that the van der Waals effect decreases for non-uniform aggregation (*F*_13;vdW_/*F*_11;vdW_ = 0.625), while the double layer effect exhibits a new exponential dependence from the formation of the particle clusters. Equations 5–11 can now be expanded in order to describe particle assembly in two dimensions.

### Aggregation in two dimensions

**Figure 3:**
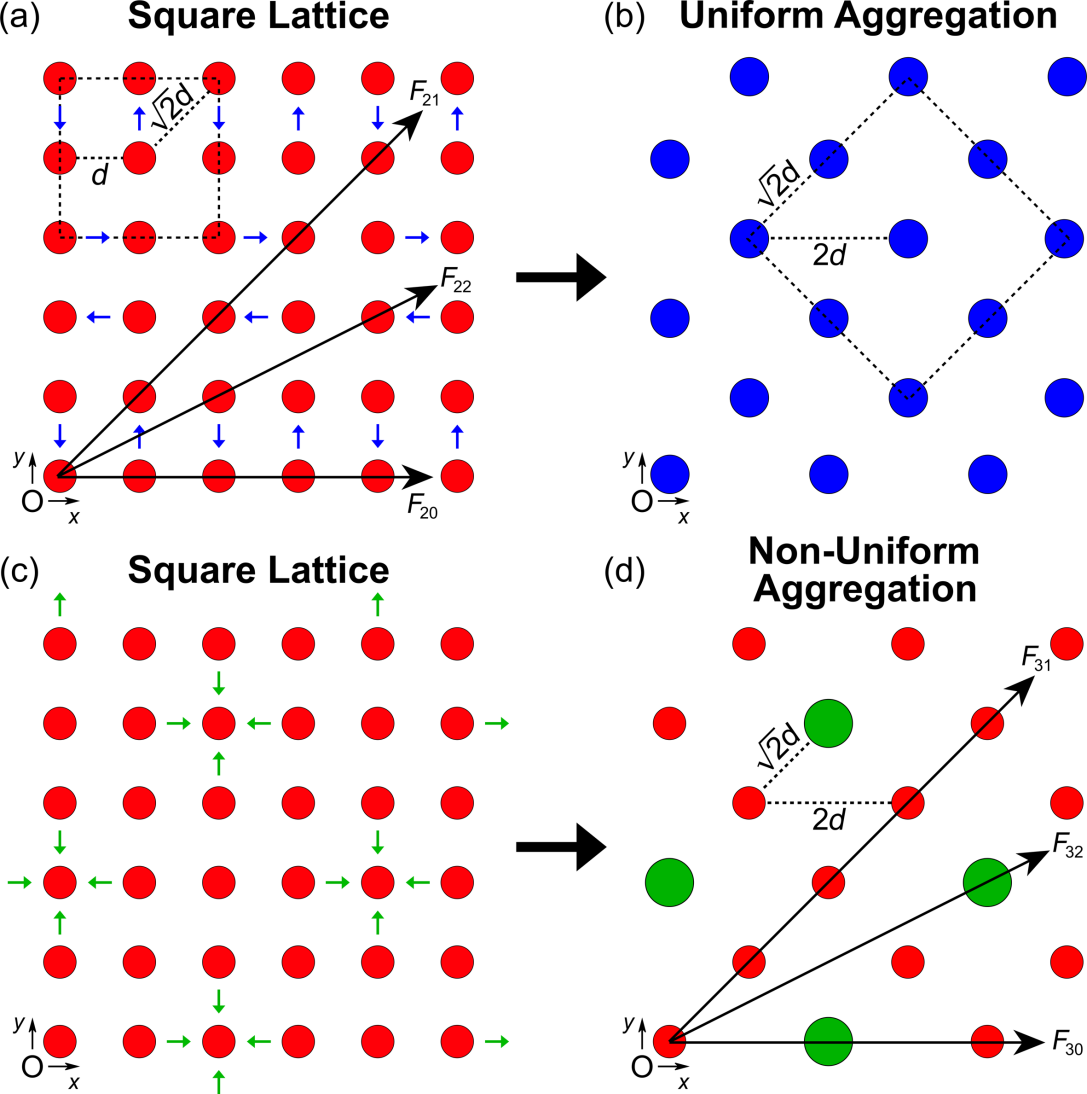
(a) Two-dimensional square lattice of particles with a uniform spacing of *d*. A single particle centered at the origin, O, feels the net force from each infinite string of particles in all directions. Particles undergo uniform aggregation when two neighboring particles combine (blue arrows). (b) The resulting uniform aggregation from (a) leads to a new square lattice of particle doublets with a uniform spacing of 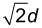. (c) Particles in a uniform square lattice undergo non-uniform aggregation when four neighboring particles aggregate about a center particle (green arrows). (d) The resulting non-uniform aggregation from (c) leads to a new lattice of five-particle clusters surrounded by singlets. A single particle at the origin feels the net force from each infinite string of clusters and singlets.

Next, we consider two variations of an infinite, two-dimensional (2D) array of particles, that is, a uniform square lattice and a hexagonal (triangular) lattice. First, we focus on the uniform square lattice (Figure 3a). The net DLVO force, *F*_2_, on a single particle positioned at the origin (O) results from the sum of each infinite string of particles acting in the positive *x*-direction. Symmetry about the *x*-axis causes the horizontal components of the force to be additive while the upward (*y* > 0) and downward (*y* < 0) vertical components of the force will cancel each other out. For example, the *x*-component of the van der Waals force along the 45° line in Figure 3a is


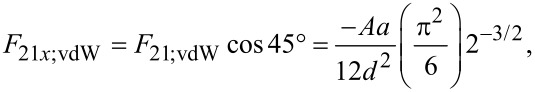


with a total contribution of 2*F*_21_*_x_*_;vdW_. Thus, *F*_2_ is given by the sum of all van der Waals and double layer contributions in the horizontal direction (*x* > 0):

[12]
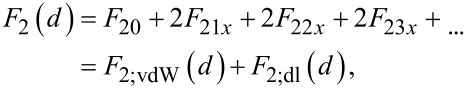


[13] $$F_{2;\text{vdW}}\left(d\right)=F_{11;\text{vdW}}\left(d\right)\left[\begin{array}{c} 1+2\sum_{n=1}^{\infty }\frac{n}{{\left(n^{2}+1\right)}^{3/2}}\\ +2\sum_{n=2}^{\infty }\frac{1}{{\left(n^{2}+1\right)}^{3/2}} \end{array}\right]$$

[14] $$F_{2;\text{dl}}\left(d\right)=F_{11;\text{dl}}\left(d\right)+2Z\frac{a}{\lambda_{\text{D}}}\left[\begin{array}{l} \sum_{n=1}^{\infty }\frac{n{\left(n^{2}+1\right)}^{-1/2}}{e^{\left(\sqrt{n^{2}+1}\right)d/\lambda_{\text{D}}}-1}\\ +\sum_{n=2}^{\infty }\frac{{\left(n^{2}+1\right)}^{-1/2}}{e^{\left(\sqrt{n^{2}+1}\right)d/\lambda_{\text{D}}}-1} \end{array}\right].$$

The first summation on the right-hand side of Equation 13 and Equation 14 accounts for the force contributions for *F*_20_ < θ < *F*_21_, while the second summation in each equation accounts for the force contributions for θ > *F*_21_, where θ is the angle of the force vector.

From this basic square lattice arrangement, the particles can again assemble by either uniform or non-uniform aggregation. When neighboring particles combine via uniform aggregation (blue arrows in Figure 3a), the result is a new square lattice of particle doublets with a uniform spacing of 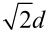 (Figure 3b) and an increase by a factor of four in the total force (*F* = 4*F*_2_(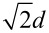)). For clarification, the pathway outlined by the blue arrows in Figure 3a is just one example of several possible uniform assembly pathways. Contrarily, when four neighboring particles combine with a center particle via non-uniform aggregation (green arrows in Figure 3c), a new lattice of five-particle clusters surrounded by singlets is formed (Figure 3d). Here, a single particle at the origin feels a net force, *F*_3_, from each infinite string of clusters and singlets, given by:

[15]
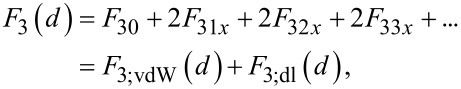


[16] $$\begin{array}{c} F_{3;\text{vdW}}\left(d\right)=F_{11;\text{vdW}}\left(d\right)[1+2\sum_{n=1}^{\infty }\frac{4n}{B^{3/2}}+2\sum_{n=1}^{\infty }\frac{2n-1}{C^{3/2}}+\\ +2\sum_{n=1}^{\infty }\frac{2}{B^{3/2}}+2\sum_{n=2}^{\infty }\frac{1}{C^{3/2}}], \end{array}$$

[17] $$\begin{array}{c} F_{3;\text{dl}}\left(d\right)=Z\frac{a}{\lambda_{\text{D}}}[f_{30;\text{dl}}\left(d\right)+2\sum_{n=1}^{\infty }\left(\frac{4n}{\sqrt{B}}\right)f_{30;\text{dl}}\left(\sqrt{B}d\right)+\\ +2\sum_{n=1}^{\infty }\frac{C^{-1/2}\left(2n-1\right)}{e^{\sqrt{C}d/\lambda_{\text{D}}}-1}+2\sum_{n=1}^{\infty }\left(\frac{2}{\sqrt{B}}\right)f_{30;\text{dl}}\left(\sqrt{B}d\right)+\\ +2\sum_{n=2}^{\infty }\frac{C^{-1/2}}{e^{\sqrt{C}d/\lambda_{\text{D}}}-1}], \end{array}$$

where


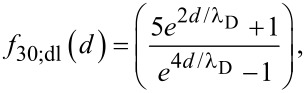


*B* = [(4*n*)^2^ + 4], and *C* = [3*^n^* + (−1)*^n^*]. Similar to the result for *F*_2_, the first and second summations on the right-hand side of Equation 16 and Equation 17 account for the force contributions from *F*_30_ < θ < *F*_31_, while the third and fourth summations in each equation account for the force contributions from θ > *F*_31_.

**Figure 4:**
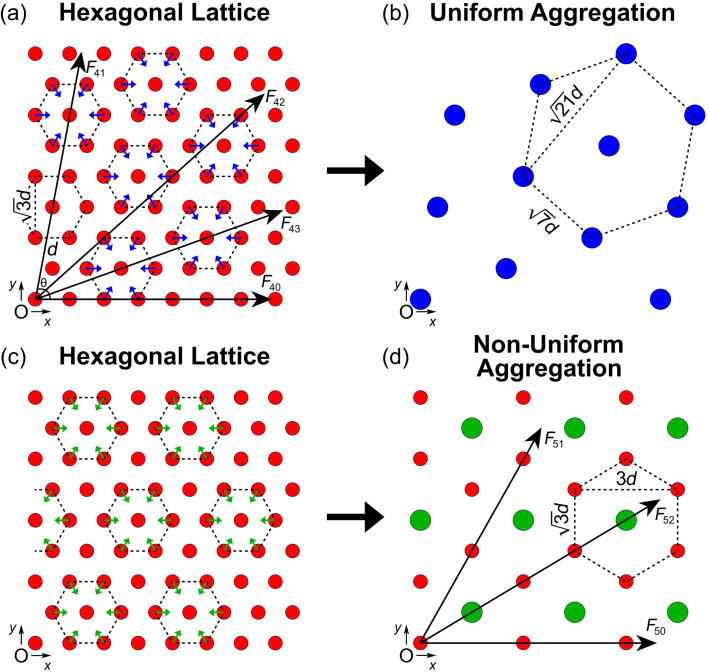
(a) Two-dimensional hexagonal lattice of particles with a uniform spacing of *d*. A single particle centered at the origin, O, feels the net force from each infinite string of particles in all directions. Uniform aggregation occurs when all particles in the lattice combine about a center particle (blue arrows). (b) The resulting uniform aggregation from (a) leads to a new hexagonal lattice of seven-particle clusters with a uniform spacing of 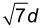. (c) Particles in a hexagonal lattice undergo non-uniform aggregation when non-neighboring particle aggregates form about a center particle (green arrows). (d) The resulting non-uniform aggregation from (c) leads to a new lattice of seven-particle clusters surrounded by single particles. A single particle at the origin feels the net force from each infinite string of clusters and singlets.

The approach outlined above can also be used for the analysis of the hexagonal lattice structure (Figure 4a) where the net DLVO force, *F*_4_, is again given by the sum of all van der Waals and double layer contributions in the horizontal direction (*x* > 0):

[18]
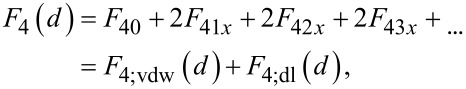


[19] $$\begin{array}{c} F_{4;\text{vdW}}\left(d\right)=F_{11;\text{vdW}}\left(d\right)[2+\frac{1}{\sqrt{3}}+2\sum_{n=3}^{\infty }\frac{2n-1}{{\left(n^{2}-n+1\right)}^{3/2}}+\\ +3\sum_{n=3}^{\infty }\frac{2n-1}{{\left(3n^{2}-3n+1\right)}^{3/2}}], \end{array}$$

[20] $$\begin{array}{c} F_{4;\text{dl}}\left(d\right)=2F_{11;\text{dl}}\left(d\right)+Z\frac{a}{\lambda_{\text{D}}}[\frac{\sqrt{3}}{e^{\sqrt{3}d/\lambda_{\text{D}}}-1}+\\ +2\sum_{n=3}^{\infty }\frac{\left(2n-1\right){\left(n^{2}-n+1\right)}^{-1/2}}{e^{\left(\sqrt{n^{2}-n+1}\right)d/\lambda_{\text{D}}}-1}+\\ +3\sum_{n=3}^{\infty }\frac{\left(2n-1\right){\left(3n^{2}-3n+1\right)}^{-1/2}}{e^{\left(\sqrt{3n^{2}-3n+1}\right)d/\lambda_{\text{D}}}-1}]. \end{array}$$

The first summation on the right-hand side of Equation 19 and Equation 20 accounts for the outer force contributions where θ < *F*_43_ and θ > *F*_42_, while the second summation in each equation accounts for the more central force contributions where *F*_43_ < θ < *F*_42_.

Particles in this hexagonal lattice configuration undergo uniform assembly when neighboring particles aggregate about a shared, central particle (blue arrows in Figure 4a). A new uniform hexagonal lattice is formed with clusters of seven particles separated by a triangular spacing of 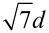 (Figure 4b), while the total force of the system increases by a factor of 49 (*F* = 49*F*_4_(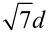)). Alternatively, non-uniform aggregation from the hexagonal lattice occurs when non-neighboring particle aggregates form (green arrows in Figure 4c) leading to a new lattice of seven-particle clusters surrounded by singlets (Figure 4d). The net force on a single particle at the origin, *F*_5_, is given by:

[21]
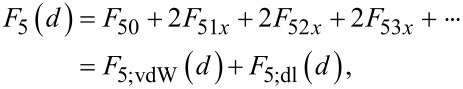


[22] $$\begin{array}{c} F_{5;\text{vdW}}\left(d\right)=\frac{-Aa}{12d^{2}}[\frac{2}{9}\cdot \frac{\pi^{2}}{6}\left(1+\sum_{n=2}^{\infty }\frac{3n-1}{P^{3/2}}\right)+\\ +\frac{8.377}{\sqrt{3}}(\frac{3}{7^{3/2}}+\sum_{n=1}^{\infty }\frac{2n-1}{P^{3/2}}+\sum_{n=2}^{\infty }\frac{6n}{Q^{3/2}}+\\ +\sum_{n=2}^{\infty }\frac{6n-4}{R^{3/2}})], \end{array}$$

[23] $$\begin{array}{c} F_{5;\text{dl}}\left(d\right)=Z\frac{a}{\lambda_{\text{D}}}[\frac{2}{e^{3d/\lambda_{\text{D}}}-1}\left(1+\sum_{n=2}^{\infty }\frac{P^{-1/2}\left(3n-1\right)}{e^{3\sqrt{P}d/\lambda_{\text{D}}}-1}\right)+\\ +\sqrt{3}(\frac{3}{\sqrt{7}}\cdot f_{50;\text{dl}}\left(\sqrt{21}d\right)+\\ +\sum_{n=1}^{\infty }\left(\frac{2n-1}{\sqrt{P}}\right)f_{50;\text{dl}}\left(\sqrt{3P}d\right)+\\ +\sum_{n=2}^{\infty }\left(\frac{6n}{\sqrt{Q}}\right)f_{50;\text{dl}}\left(\sqrt{3Q}d\right)+\\ +\sum_{n=2}^{\infty }\left(\frac{6n-4}{\sqrt{R}}\right)f_{50;\text{dl}}\left(\sqrt{3R}d\right))], \end{array}$$

where


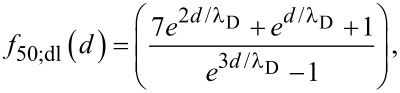


*P* = [3*n*^2^ − 3*n* + 1], *Q* = [(3*n*)^2^ − (3n − 1)], and *R* = [(3*n* − 2)^2^ − (3*n* − 3)]. Here, the first summation on the right-hand side of Equation 22 and Equation 23 accounts for the force contribution from each infinite string of single particles (cf. *F*_51_, Figure 4d). The second, third, and fourth summations in each equation account for the force contributions from each infinite string of alternating singlets and clusters (cf. *F*_52_, Figure 4d).

Equations 12–23 can now be used to predict the effects of varying system parameters on the particle assembly behavior. It should be noted that Figures 2–4 are merely snapshots of a transient system – not the final state of the system –, which will continue to evolve and form new aggregates until equilibrium is reached. The approaches outlined above are recursive and can continue to be carried out as the particle spacing and aggregate size changes.

## Results and Discussion

The constructal model outlined above provides a useful framework for predicting, tuning, and optimizing colloidal particle assembly. As a proof of concept, we start with a representative particle/solvent combination, where *a*_0_ = 25 nm, ζ_p_ = −25 mV, *I* = 0.011 M, and ε_c_ = 80 (water), with an initial particle lattice spacing of *d* = 5 nm. The effect a given system parameter has on the overall mechanism of assembly with respect to lattice configuration can now be evaluated by simply adjusting the desired parameter while holding the others constant.

**Figure 5:**
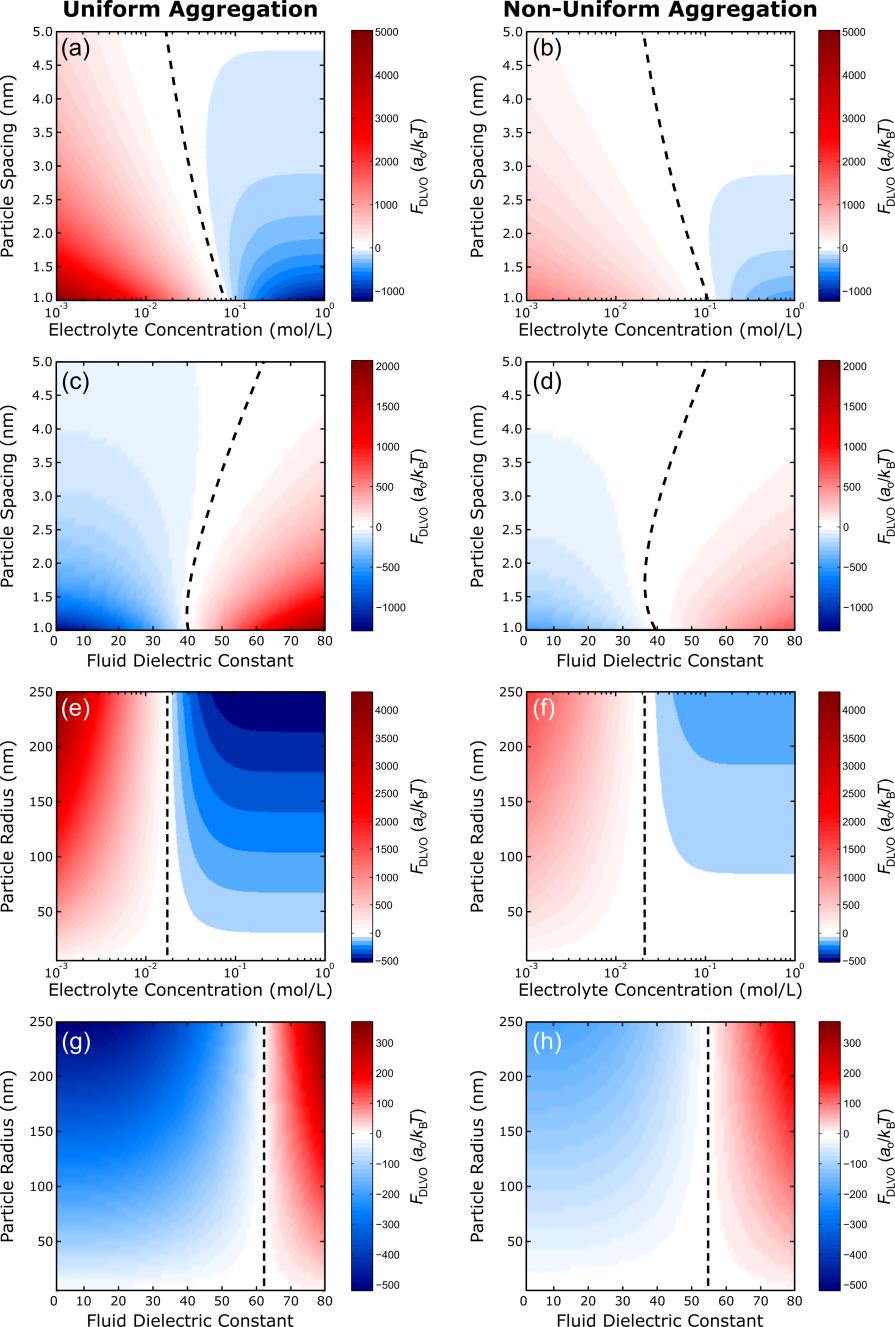
Square lattice. Surface plots of the predicted DLVO force for both uniform (left) and non-uniform (right) aggregation of a square lattice as a function of (a–d) particle spacing or (e–h) particle radius and (a,b,e,f) electrolyte concentration or (c,d,g,h) fluid dielectric constant. The dashed lines show the location of the stable fixed points where the net force is zero. Positive values (red) indicate repulsive regions while negative values (blue) indicate attractive regions. Parameters: (a–d) *a*_0_ = 25 nm, (e–h) *d* = 5 nm, ζ_p_ = −25 mV, (a,b,e,f) ε_c_ = 80, and (c,d,g,h) *I* = 0.011 M.

**Figure 6:**
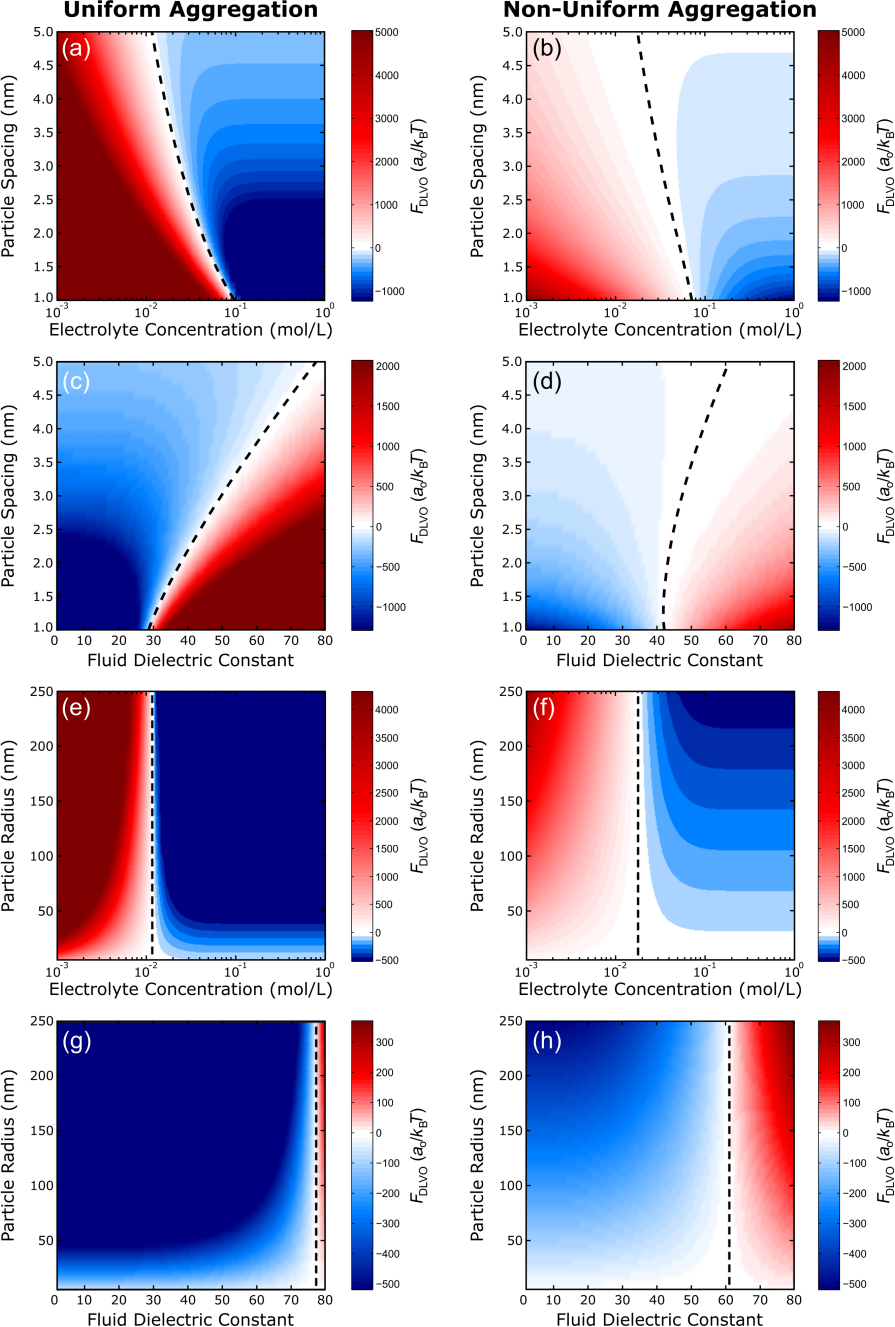
Hexagonal lattice. Surface plots of the predicted DLVO force for both uniform (left) and non-uniform (right) aggregation of a hexagonal lattice as a function of (a–d) particle spacing or (e–h) particle radius and (a,b,e,f) electrolyte concentration or (c,d,g,h) fluid dielectric constant. The dashed lines show the location of the stable fixed points where the net force is zero. Positive values (red) indicate repulsive regions while negative values (blue) indicate attractive regions. Parameters: (a–d) *a*_0_ = 25 nm, (e–h) *d* = 5 nm, ζ_p_ = −25 mV, (a,b,e,f) ε_c_ = 80, and (c,d,g,h) *I* = 0.011 M.

The predicted results of the square and hexagonal lattice arrangements are shown in Figure 5 and Figure 6, respectively. The total DLVO forces from both uniform (left) and non-uniform (right) aggregation are plotted as functions of particle spacing or particle radius and electrolyte concentration or fluid dielectric constant. The same general trends were observed for both lattice configurations and are describe herein. Figure 5a,b and Figure 6a,b show the results of varying particle spacing as a function of the electrolyte concentration. Here, the magnitude of the total DLVO force is represented by the color intensity while the color itself represents whether attractive or repulsive forces dominate. Red indicates areas where the repulsive double layer force dominates while blue indicates areas where the attractive van der Waals force dominates. When the double layer and van der Waals forces are equal, the total DLVO force is zero leading to a stable fixed point (cf. Figure 1b). The dashed lines in Figure 5 and Figure 6 denote the locations of these stable fixed points. As expected, when the particles are moved closer together the magnitude of the DLVO force increases. Likewise, as the electrolyte concentration is increased (for both uniform and non-uniform aggregation) the particles become less repulsive and transition to an attractive regime where aggregation is favorable. Figure 5c,d and Figure 6c,d show a similar result for the effect of particle spacing as a function of the fluid dielectric constant such that lower dielectric solvents lead to an increased attractive force and particle aggregation.

The effect of the particle radius on the assembly behavior in each lattice arrangement, with respect to the solvent properties, is shown in Figure 5e–h and Figure 6e–h. As expected from Equation 1 and Equation 2, the size of the particle only influences the magnitude of the DLVO force, which scales linearly with particle radius, that is, larger particles are more attractive/repulsive. Ultimately, only the solvent properties are responsible for determining whether or not aggregation will be favorable. Similar to varying the particle spacing, aggregation is most favorable at high salt concentrations (Figure 5e,f and Figure 6e,f) and low dielectric constants (Figure 5g,h and Figure 6g,h). This trend can be explained by taking a closer look at the definition of the Debye length [26]:

[24] $$\lambda_{\text{D}}=\sqrt{\frac{\varepsilon_{0}\varepsilon_{\text{c}}k_{\text{B}}T}{2N_{\text{A}}e^{2}I}},$$

where *N*_A_ is Avogadro’s number, and *e* is the elementary charge. A sufficiently thin double layer will allow the particles to come into closer contact where van der Waals attraction can occur. Consequently, increasing the electrolyte concentration or decreasing the fluid dielectric constant will decrease the Debye length and promote aggregation.

The results in Figure 5 and Figure 6 reveal that the magnitude of the total DLVO force, regardless of nature (attractive or repulsive), is expected to be greater for uniform aggregation than for non-uniform aggregation. The nature of this force does, however, naturally influence particle behavior. For example, at a fluid dielectric constant value of ε_c_ = 10, the total DLVO force is negative (Figure 5c,d,g,h and Figure 6c,d,g,h). Since the magnitude of this force is greater in the uniform scenario than in the non-uniform scenario, uniform aggregation is expected. For the square lattice, specifically, this increased magnitude is accompanied by a shift in the location of the stable fixed points, which suggests that uniform aggregation of particles is typically preferred with respect to van der Waals and double layer interactions. For comparison, a value of ε_c_ = 80 yields a positive total DLVO force and separation is expected. Since the magnitude of this force is smaller for the non-uniform scenario, the system is “less repulsive” such that a non-uniform pathway could potentially be more conducive for aggregation.

Unlike the square lattice, where predicted particle movement (aggregation/separation) is the same regardless of the assembly mechanism (uniform/non-uniform), the hexagonal lattice shows regimes where the assembly mechanism seems to influence the particle behavior. For example, when the particles are in close proximity (*d* = 1 nm) at an electrolyte concentration of *I* = 0.08 M (Figure 6a,b) or a fluid dielectric constant value of ε_c_ = 35 (Figure 6c,d), the uniform scenario leads to a net repulsive force. Conversely, for the non-uniform scenario these system parameters predict a net attractive force. Therefore, the solvent properties could be tuned by changing either the salt concentration or dielectric constant in order to induce a desired assembly mechanism. Moreover, the overall magnitudes of the DLVO forces in the hexagonal lattice (Figure 6) are greater than the magnitudes of the forces in the square lattice (Figure 5), that is, the driving force for aggregation (or separation) is greater for the hexagonal system. In the square lattice structure, a single particle is surrounded by four neighbors within one *d*-spacing, while a particle in the hexagonal lattice structure is surrounded by six neighbors within one *d*-spacing. Thus, the increase in driving force for the hexagonal system is likely due to this increase in the coordination number.

We should note that the results presented here assume the self-assembly of monodisperse spherical particles suspended in a monovalent salt solution arranged in a uniform starting configuration. Given the Brownian nature of colloidal particles, future simulations should account for the randomness of colloidal suspensions. These simulations should also include the effects from polydisperse particle mixtures or divalent salt solutions, and they should be able to handle the addition of an external stimulus that could facilitate and drive particle assembly.

## Conclusion

We have developed the first model based on constructal theory to explain the self-assembly mechanisms of charged colloidal particles. This theoretical model, based on traditional DLVO interactions, can be broadly applied to many colloidal systems and is used here to predict the particle aggregation and equilibrium behavior as a function of varying system parameters. Our modeling results indicate that uniform aggregation of a square lattice is preferred over non-uniform aggregation with respect to the overall magnitude of the total DLVO force. When arranged in a hexagonal lattice, however, the preferred mechanism of assembly is determined by the solution properties and can be tuned by varying the salt concentration or fluid dielectric constant. Possible expansions to this theory include accounting for various particle mixtures, solvent properties, and particle configurations, as well as adding external driving forces (i.e., electric or magnetic fields) and, finally, a third dimension. Nonetheless, our model provides a fundamental framework for understanding and tuning the assembly behavior of colloidal particles and can have broad implications on fabrication techniques.
